# Supplementary material for: Mechanisms governing the pioneering and redistribution capabilities of the non-classical pioneer PU.1
Source: Nat Commun. 2020 Jan 21;11:402. doi: 10.1038/s41467-019-13960-2 (PMC6972792; doi:10.1038/s41467-019-13960-2)
Supplement: Supplementary file 7 — Source data [file 41467_2019_13960_MOESM7_ESM.zip › Source_Data/Figure6/Figure6B_MotifScanOutput/FLI1_PU.1/homerResults/motif44.similar.html]

motif44

## Information for motif44

G
A
C
T
T
G
A
C
A
G
C
T
A
G
T
C
A
C
T
G
A
G
T
C
A
C
T
G
G
T
C
A
A
C
T
G
T
C
G
A
G
T
C
A
A
T
G
C
  
Reverse Opposite:  

T
A
C
G
C
A
G
T
A
G
C
T
T
G
A
C
C
A
G
T
G
T
A
C
C
T
A
G
A
G
T
C
A
C
T
G
T
C
G
A
A
C
T
G
C
T
G
A
  

|  |  |
| --- | --- |
| p-value: | 1e-18 |
| log p-value: | -4.374e+01 |
| Information Content per bp: | 1.697 |
| Number of Target Sequences with motif | 82.0 |
| Percentage of Target Sequences with motif | 0.82% |
| Number of Background Sequences with motif | 65.9 |
| Percentage of Background Sequences with motif | 0.18% |
| Average Position of motif in Targets | 102.3 +/- 58.6bp |
| Average Position of motif in Background | 97.8 +/- 70.9bp |
| Strand Bias (log2 ratio + to - strand density) | 0.1 |
| Multiplicity (# of sites on avg that occur together) | 1.18 |
| Motif File: | file (matrix) reverse opposite |

### Similar de novo motifs found

|  |  |  |  |  |  |  |  |
| --- | --- | --- | --- | --- | --- | --- | --- |
| Rank | Match Score | Redundant Motif | P-value | log P-value | % of Targets | % of Background | Motif file |
| 1 | 0.979 | G T C A A G C T T G A C A C G T G T A C A C T G A G T C A C T G C T G A C A T G T C G A | 1e-15 | -35.955065 | 0.76% | 0.19% | motif file (matrix) |
| 2 | 0.937 | A C G T G T A C A C G T A G T C A C T G A G T C A C T G C G T A | 1e-13 | -30.692327 | 0.69% | 0.18% | motif file (matrix) |
| 3 | 0.934 | T G C A G A C T G T A C A G C T G T A C C A T G A G T C A C T G C T G A C T A G | 1e-12 | -28.313858 | 0.50% | 0.11% | motif file (matrix) |
